# Supplementary material for: Frequency, timing and risk factors for primary maternal cytomegalovirus infection during pregnancy in Quebec
Source: PLoS One. 2021 Jun 25;16(6):e0252309. doi: 10.1371/journal.pone.0252309 (PMC8232530; doi:10.1371/journal.pone.0252309)
Supplement: S1 Table — (DOCX) [file pone.0252309.s001.docx]

S1 Table. Distribution of clinical and sociodemographic characteristics in the study participants compare to the whole “grossesse en santé” cohort

|  | **Study population n=4,111** | | **“Grossesse en santé” (original sample) n= 7,855** | |  |
| --- | --- | --- | --- | --- | --- |
| **Variables** | **n** | **Frequency** | **n** | **Frequency** | **P-value** |
| **Age (years)** | 4111 |  | 6984 |  | 0.583 |
| 18–24 | 607 | 14.7 | 974 | 13.9 |  |
| 25–29 | 1749 | 42.5 | 2956 | 42.3 |  |
| 30–34 | 1322 | 32.1 | 2310 | 33.0 |  |
| 35–45 | 433 | 10.3 | 744 | 10.6 |  |
| **Parity** | 4111 |  | 7859 |  | 0.636 |
| 2 children or more | 529 | 12.8 | 1058 | 13.4 |  |
| 1 child | 1610 | 39.1 | 3076 | 39.1 |  |
| 0 (nulliparous) | 1972 | 47.9 | 3725 | 47.4 |  |
| **Annual household income** | 3528 |  | 6449 |  | <0.0001 |
| Less de 15,499 $ (4) | 124 | 3.5 | 240 | 3.7 |  |
| 15,500 $- 24,999 $ (3) | 201 | 5.7 | 399 | 6.1 |  |
| 25,000 $- 39,999 $ (2) | 466 | 13.2 | 841 | 13.0 |  |
| 40,000 $- 59,999 $ (1) | 777 | 22.0 | 1426 | 22.1 |  |
| 60,000 $ and above (0) | 1960 | 55.5 | 3543 | 54.9 |  |
| **Ethnicity** | 3675 |  | 6741 |  | 0.631 |
| Others (African-Canadian, Asian, Latin-Canadian, Canadian First Nation and others) | 121 | 3.2 | 234 | 3.4 |  |
| Caucasian | 3554 | 96.7 | 6507 | 96.5 |  |
| **Marital status** | 3793 |  | 10752 |  | 0.793 |
| Single | 263 | 6.9 | 472 | 6.7 |  |
| Married | 837 | 22.0 | 1581 | 22.7 |  |
| Separated/Divorced | 26 | 0.6 | 55 | 0.7 |  |
| Common-law partner | 2667 | 70.3 | 4851 | 69.7 |  |
| **County of birth** | 3809 |  | 7007 |  | 0.280 |
| Canada or United States | 3665 | 96.2 | 6712 | 95.7 |  |
| Others countries | 144 | 3.7 | 295 | 4.2 |  |
| **Work (part-time or full-time)** | 2573 |  | 4708 |  | 0.217 |
| Yes | 2347 | 91.2 | 4253 | 90.3 |  |
| No | 226 | 8.7 | 455 | 9.6 |  |
| **Level of education** | 3794 |  | 6959 |  | 0.704 |
| None (high school not completed) | 147 | 3.8 | 293 | 4.2 |  |
| Secondary (including professional) | 894 | 23.5 | 1656 | 23.8 |  |
| College (CEGEP) | 1280 | 33.7 | 2285 | 32.8 |  |
| University level | 1473 | 38.8 | 2725 | 39.1 |  |
