# Supplementary material for: Frequency, timing and risk factors for primary maternal cytomegalovirus infection during pregnancy in Quebec
Source: PLoS One. 2021 Jun 25;16(6):e0252309. doi: 10.1371/journal.pone.0252309 (PMC8232530; doi:10.1371/journal.pone.0252309)
Supplement: S1 File — (DOCX) [file pone.0252309.s002.docx]

**Codebook**

File name: extraction projet PR67F Ig G et Ig M

Prenom= id du patient( removed)

**Test:**

cmvg2= Ig G

Cmvm2=Ig M

**Resultat** = estimation

**Avidity test**

File name: PR67F 2019-04-26 avidite

CMVA = avidity of Ig G
