# Supplementary material for: Frequency, timing and risk factors for primary maternal cytomegalovirus infection during pregnancy in Quebec
Source: PLoS One. 2021 Jun 25;16(6):e0252309. doi: 10.1371/journal.pone.0252309 (PMC8232530; doi:10.1371/journal.pone.0252309)
Supplement: Attachment — Submitted filename: Response to reviewers.docx [file pone.0252309.s005.docx]

Dr. Juliet Spencer

Academic Editor

PLOS ONE

March 23, 2021

Dear Dr. Spencer:

We are pleased that our manuscript entitled, “Frequency, timing and risk factors for primary maternal cytomegalovirus infection during pregnancy in Quebec” received overall positive review by your editorial team and we thank Reviewers 1 and 2 for their helpful comments. These comments have been addressed individually (see Word™ document entitled “Main document_PLOS one final ”) as described below. We hope that in its revised form our manuscript meets the standards for publication in PLOS ONE.

Yours sincerely,

Dr. Isabelle Boucoiran

____________________________________________________________________________________

**REVIEWER COMMENTS AND ACTIONS TAKEN**

**Reviewer #1**:

1) **For Table 1 it might be helpful if the authors could explain in the text or the materials and methods section what their abbreviations on top of each row mean and what the difference between their unadjusted and adjusted analysis is.**

The column headings in Table 1 have been clarified. Details regarding unadjusted and adjusted analyses have been provided in “Materials and Methods” (see lines 136-141, 151-154,).

2) **Similarly, for table 2 further explanations on what the rate and what the unadjusted rate is are needed. Also, there are * and ** after Rate and Unadjusted RR, but I can’t find what these asterisks refer to.**

The column headings in table 2 have been modified for clarity and asterisks are defined at the bottom of the table. The unadjusted rate ratio is explained in “Materials and Methods” (lines 152 – 154).

3) **I can’t find figure legends. Figure 1 is probably fine, but figure 2 and 3 might benefit from some figure legends explaining what is shown.**

Legends to the Figures have been added (see Results).

4) **There are some formatting issues with missing gaps or additional full stops in lines 135, 201, 214, 222, 224 and 232**

Formatting issues have been addressed.

**Reviewer #2 :**

1) **The authors should please add original data from their seroepidemiological study, like IgM indices, IgG levels and IgG avidity assays. This could help to understand the different subcohorts of the seropositive pool of 965 mothers.**

Original data from our seroepidemiological study data (IgM indices, IgG levels and IgG avidity assays) have been included as a part of this resubmission (see attached excel files of data: 1) extraction projet PR67F Ig G et Ig M and 2) PR67F 2019-04-26 avidite).

2) **Why in the seroconversion data in T3 of primary infections no CMV IgG avidity data are given? It would also be helpful to see the original data of CMV IgG levels, IgM indices, and CMV IgG avidity.**

The purpose of Ig G avidity analysis is to distinguish between recent and past infection. T3 blood sample analyses enabled us to establish the frequency of primary infection occurring through IgG seroconversion between T1 and T3. The authors feel that additional avidity analyses are not warranted.

3) **These lacking data should-if available- also be evaluated statistically to characterise CMV primary infections versus non-primary infections or latent CMV infections. Do all primary infected women in T3 really express clear low IgG avidity?**

We did not have data for T3 CMV IgG avidity to characterize primary versus other CMV infection. Rather, we used IgG seroconversion, which is a better indicator of recent infection than avidity. Standard serological testing and avidity cannot distinguish for non-primary infection.

4) **Fig 2: how the drop in CMV seroconversion from 2005 to 2006 can be explained? Is it a potential bias, since in 2005 only 185 pregnant women were included?**

We believe Reviewer 1 is referring to seroprevalence rather than seroconversion. We do not know why seroprevalence was higher in 2005 than during the rest of the study duration and have changed our manuscript to reflect this (lines 180-182). Irrespective of this “outlier” changepoint analysis revealed no observable trends in seroprevalence over the study, including between years 2005 and 2006.
